# Infant Feeding Practices in China and Ireland: Ireland Chinese Mother Survey

**DOI:** 10.3389/fpubh.2018.00351

**Published:** 2018-11-28

**Authors:** Qianling Zhou, Katherine M. Younger, John M. Kearney

**Affiliations:** ^1^Department of Maternal and Child Health, School of Public Health, Peking University, Beijing, China; ^2^School of Biological Sciences, Dublin Institute of Technology, Dublin, Ireland

**Keywords:** breastfeeding, Chinese, Ireland, migration, infant feeding

## Abstract

**Introduction:** Migration to another country may induce changes in infant feeding practices especially where such practices differ considerably between the two countries. This study was undertaken to compare the infant feeding practices between Chinese mothers who gave birth in Ireland (CMI) with immigrant Chinese mothers who gave birth in China (CMC), and to examine the factors that influence these practices.

**Methods:** A cross-sectional self-administrated survey was conducted among a convenience sample of 322 Chinese mothers living in Ireland. Data were obtained from mailed questionnaires. Infant feeding practices between CMC and CMI were compared by Chi-square or independent sample *t*-test. Binary logistic regression analyses were further performed to test the differences in infant feeding practices between two groups, after controlling for potential socio-demographic confounders.

**Results:** High breastfeeding initiation rates were found in both groups (CMC: 87.2%; CMI: 75.6%); however sharp reductions in breastfeeding rates at 3 months (49.1%) and 6 months (28.4%) were found among CMI but not CMC (*P* < 0.05). Introduction of water within 1 week after childbirth was common for CMC in comparison with CMI. CMI were more likely than CMC to introduce infant formula to their child within the first 4 months after childbirth. The timing of introduction of rice porridge, vegetables, fruits and meats did not differ between CMC and CMI.

**Conclusions:** Cultural and perceptional factors, and changes caused by migration contribute to the decline in breastfeeding duration among CMI. Language-specific breastfeeding support and education among Chinese mothers in Ireland is needed, in particular to encourage mothers to breastfeed for 6 months or more.

## Introduction

Factors associated with infant feeding practices, including maternal demographic, social, cultural and psychological, have been extensively explored (1, 2). Migration to another country may induce changes in infant feeding practices as the social environment and maternal socio-demographic status differ. A decrease in breastfeeding rates has been commonly reported in some Asian immigrant groups living in Western countries (3–7), which has been attributed to the transition from an extended to a nuclear family (8), an increased interest in Western norms (8), a need to work or study (8, 9), the social isolation caused by language barriers (6, 10), the availability of infant formula (11), an inability to access and consume traditional confinement foods (4, 12), conflict between their traditional practices and those of the host culture (13) and acculturation (14).

While the majority of mothers in China choose to breastfeed (15), previous studies found that immigrant Chinese mothers in Europe or North America seldom nursed their children (10, 16). Although the breastfeeding initiation rate remains high for Chinese mothers living in Australia, shorter breastfeeding duration was revealed (17). The perceptions of inadequate breast milk (5, 17) and inconvenience of breastfeeding, and the use of traditional infant solids are prevalent among the Chinese immigrants (18, 19).

Ireland's breastfeeding initiation rate is among the lowest in the world (20–22); despite some gradual increases over the last 10 years (23). Low rates of exclusive breastfeeding at hospital discharge (46.3%) (22) and at 6 months (15%) (24), and early introduction to complementary foods (before 12 weeks) (25) are reported. A 5-year breastfeeding action plan (2016–2021) has recently been published to improve breastfeeding rates and support mothers to breastfeed in Ireland (26). The population of non-nationals in Ireland has increased by 87% from the year 2002 (224,261 persons) to 2006 (419,733 persons). The number then stabilized at 544,357 persons in 2011 (27); but the most recent census data suggest these figures are rising again (28). A national infant feeding survey has suggested a need to understand the infant feeding practices of non-Irish women in Ireland (29). Hence, this project was undertaken to fill an information gap, to acquire an insight into the infant feeding practices of the Chinese, who represent one of the largest ethnic groups in Ireland (28, 30), and to determine to what extent maternal infant feeding practices have been influenced by migrating to Ireland. We compared Chinese mothers who gave birth in China (CMC) and Chinese mothers who gave birth in Ireland (CMI) in terms of their (1) breastfeeding rates, (2) milk feeding and weaning practices, and (3) potential influences of infant feeding.

## Materials and methods

### Participants

Participants were Chinese women who were born in China (including Hong Kong and Macau), had given birth to at least one child, and had been in Ireland for at least 6 months.

### Data collection

This study was advertised in a local Chinese newspaper and websites which were well-known among the Chinese in Ireland, and was announced in several Chinese communities in Ireland. Potential participants were approached at places frequented by Chinese mothers in Dublin and suburban areas, including Chinese supermarkets, Chinese weekend language schools for children, Chinese cultural music and dancing school, church organizations and Chinese restaurants, between September and December 2008. The purpose and demands of the study were explained to each potential participant, and mothers were assured of anonymity and confidentiality. The questionnaires were returned by mail and a follow-up telephone call was made to participants who failed to complete the questionnaire or who provided unclear information. A 5-euro shopping voucher was posted to each participant upon completion of the study. A “snowball” technique was used to increase the sample size, *i.e*. participants were required to help announce and distribute the survey to those who were known to them and met the inclusion criteria. This study was approved by the Research Ethics Committee of the Dublin Institute of Technology. Signed informed consent was obtained from all participants. Written permission to approach mothers was sought from the Chinese supermarket, restaurants, schools and church organizations. Informed consent was obtained from all individual participants included in the study.

### Measures

A cross-sectional questionnaire was devised to seek retrospective information on mothers' infant feeding practices of their youngest child (index child), and to explore the influences on practices. Participants were asked to indicate the first liquid and solid they provided to the index child, and the timing of the introduction of a number of liquids and complementary foods. Mothers were asked to recount the reasons for their feeding decisions. Mothers who breastfed the index child were also required to report the planned and actual duration of breastfeeding and to render the reasons for breastfeeding cessation. Socio-demographic information (including mother's age at time of birth, marital status, birthplace, duration in Ireland, education, youngest child's birthplace and order) and potential influences on feeding practices (newborn's sleeping place, mother's delivery mode, source of support and influences of infant feeding, length of maternity leave, maternal beliefs and consumption of the traditional post-partum diet) were collected.

The questionnaire took approximately 30 min to complete. It was reviewed for content validity, reliability and cultural appropriateness by two breastfeeding specialists and a Chinese medical doctor. It was translated into Chinese and blind back-translated to check the accuracy of the translation (7). The questionnaire (Chinese) was pilot tested on 20 Chinese mothers in Ireland to assess clarity, redundancy and language adequacy.

### Data analyses

The participants were separated into two groups: Chinese immigrant mothers who gave birth to the index child in China (CMC) vs. Chinese immigrant mothers who gave birth to the index child in Ireland (CMI). Sample size was calculated according to Daly and Bourke (31) to ensure adequate numbers of participants in each group. It was estimated that the size of groups had 80% power to detect a difference. Pearson Chi-squared tests were conducted to assess the differences of infant feeding and their influential factors between CMC and CMI. Difference in the average timing of individual solids introduction was compared by an independent sample *t*-test. Binary logistic regression analyses were further conducted to test the differences in infant feeding practices between CMC and CMI, after controlling for potential confounders. Socio-demographic variables including maternal age at time of childbirth, mothers' duration in Ireland, child's order, child's age, maternal education level, marital status and maternal birthplace were adjusted in each regression model. Data analyses were conducted with SPSS (version 15). The level of 5% significance was used in statistical analyses.

In this paper, breastfeeding is taken to mean “any breastfeeding,” i.e., the child has received breast milk with or without other drink, formula or other infant food (15). Breastfeeding initiation was defined as mother put their baby to the breast at least once and gave any breast milk (32).

## Results

### Sample characteristics

A total of 343 questionnaires were collected. With an exclusion of those who had not delivered their baby in China or Ireland, the final sample population was 322, including 47 CMC and 275 CMI. The majority of mothers were married, had a third level education (i.e., completed undergraduate and/or postgraduate study), and had been born in the northern part of mainland China. Three-fifths of CMC gave birth between 20 and 25 years old, whereas almost 70% of CMI were over 25 years old. The majority of CMC had been in Ireland for no more than 5 years however nearly 60% of CMI had been in Ireland between 5 and 10 years. Over 95% of CMC were primiparous compared with only 62.2% of CMI. The average age of children born in China was 11.72 (S.D. 5.65) years old while children born in Ireland were on average 4.11 (S.D. 3.46) years old (Table 1).

**Table 1:** Demographic characteristic of the samples.

	**Total population**	**CMC**	**CMI**
	**(*n* = 322)**	**(*n* = 47)**	**(*n* = 275)**
	**No. (%)**	**No. (%)**	**No. (%)**
Maternal age (years)^*^			
20–25	108 (34.4)	27 (60.0)	81 (30.1)
26–30	121 (38.5)	10 (22.2)	111 (41.3)
>30	85 (27.1)	8 (17.8)	77 (28.6)
**Marital status**			
Married	275 (85.4)	43 (91.5)	232 (84.4)
Single/Divorced/Widow	47 (14.6)	4 (8.5)	43 (15.6)
**Education level**	
Primary/Secondary	156 (48.4)	23 (48.9)	133 (48.4)
Tertiary	166 (51.6)	24 (51.1)	142 (51.6)
**Mother's birthplace**			
Mainland China (north)	163 (50.9)	20 (42.6)	143 (52.4)
Mainland China (south)	110 (34.4)	19 (40.4)	91 (33.3)
Hong Kong	47 (14.7)	8 (17.0)	39 (14.3)
Duration in Ireland (years)^*^			
≤5	98 (30.4)	29 (61.7)	69 (25.1)
>5–10	180 (55.9)	17 (36.2)	163 (59.3)
>10	44 (13.7)	1 (2.1)	43 (15.6)
Child's order^*^			
1	216 (67.1)	45 (95.7)	171 (62.2)
2/3/4	106 (32.9)	2 (4.3)	104 (37.8)
	Mean ± S.D.	Mean ± S.D.	Mean ± S.D.
Child's age at time of the study (years)^*^	5.22± 4.68	11.72 ± 5.65	4.11 ±3.46

*CMC, Chinese mother gave birth in China; CMI, Chinese mother gave birth in Ireland*.

*Columns where the numbers do not add up to the specific n reflect missing values for this column*.

* *P < 0.05, Pearson Chi-squared or independent sample t-test used to detect the differences between CMC and CMI*.

*Mainland China (north) included the provinces of Anhui, Hebei, Heilongjiang, Henan, Hubei, Jiangsu, Jilin, Liaoning, Shandong, Shanxi; Xinjiang Uygur Autonomous Region; and the municipality of Beijing, Tianjin*.

*Mainland China (south) included the provinces of Fujian, Guangdong, Jiangxi, Sichuang, Yunnan, Zhejiang; Guangxi Zhuang Autonomous Region; and the municipality of Chongqing, Shanghai*.

*Hong Kong indicated Hong Kong Special Administrative Regions of China. In this study, one subject from Macau was grouped into this category*.

### Breastfeeding rates between CMC and CMI

**Figure 1:**
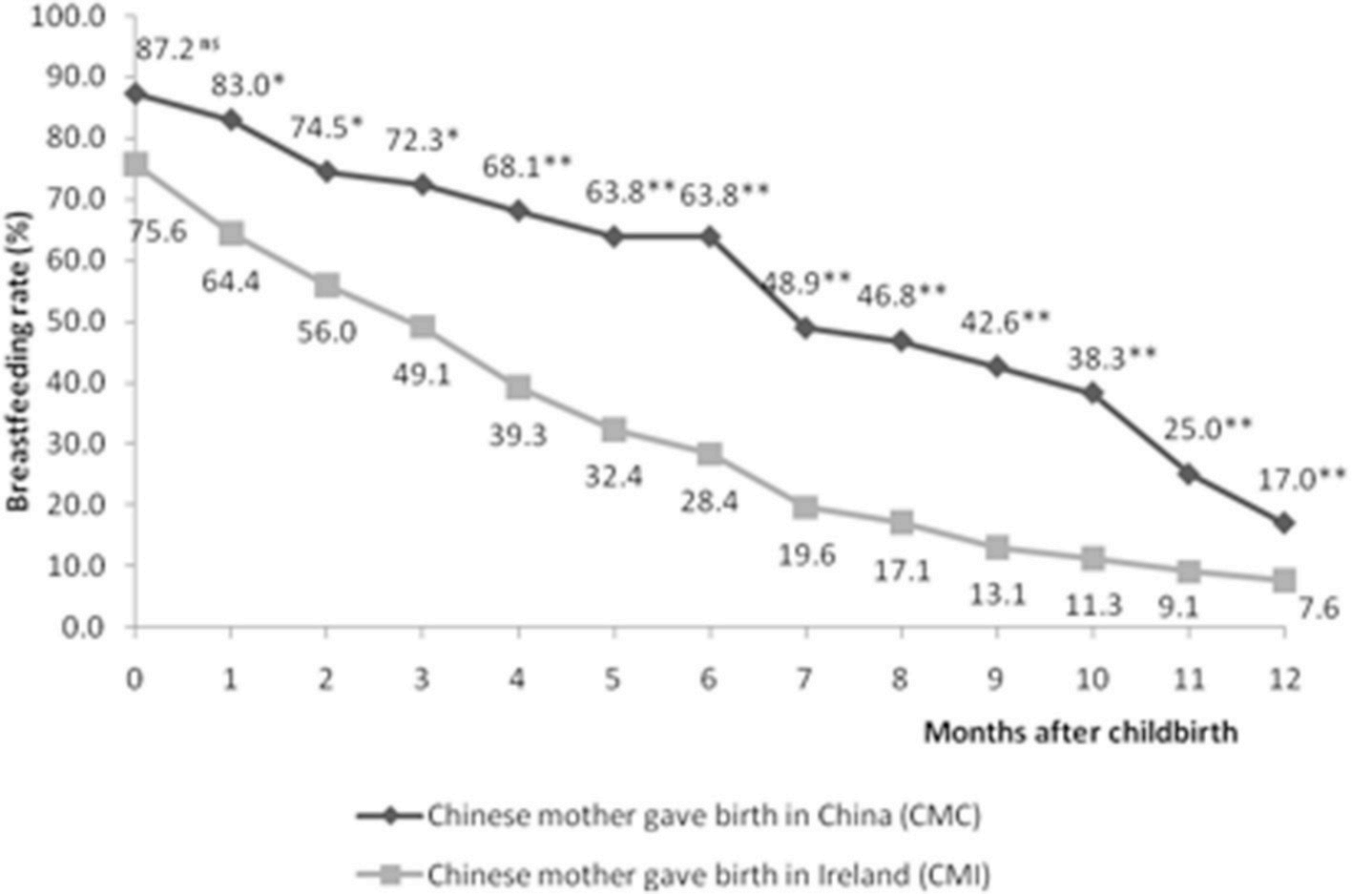
Breastfeeding prevalence between CMC (*n* = 47) and CMI (*n* = 275) at each month from birth to 12 months of life. ^*^P < 0.05; ^**^P < 0.001; ns: no significant difference. Significance of relationships was calculated by Pearson Chi-squared tests.

Figure 1 illustrates the “any breastfeeding” rates between CMC and CMI, from childbirth to 12 months of age. Initially, 41 out of 47 CMC (87.2%) and 208 out of 275 CMI (75.6%) breastfed their child. Rates of CMI dropped to 49.1% at 3 months and 28.4% at 6 months whereas the rate among CMC at 6 months remained above 60%. At 12 months, breastfeeding rates of CMC and CMI fell to 17 and 7.6%, respectively. Significant differences in breastfeeding rates between CMC and CMI were revealed from 1 to 3 months (*P* < 0.05), and even more distinct differences were found from 4 to 12 months (*P* < 0.001).

### Differences in infant feeding patterns

Univariate analyses revealed some differences in infant feeding practices between CMC and CMI. Most children born in China (44.7%) and Ireland (52.0%) were fed with breast milk first after birth. Breast milk was introduced sooner among CMI than CMC. For mothers who did not breastfeed at first, CMI mainly used infant formula (45.5%) while CMC mainly used non-milk liquids. More than 90% of infants born in either China or Ireland were given water before 4 months. The use of other non-milk-liquids (e.g., juice, tea) before 4 months was more prevalent among CMC (>62%) than CMI (<20%). The majority of CMI (>80%) introduced infant formula before 4 months, compared with 46.8% of CMC. Early introduction of cow's milk (<12 months) was found among 31.8% CMC and 8.5% of CMI. Over 60% of the children were fed according to a scheduled routine, with no difference between CMC and CMI (Table 2).

**Table 2:** Differences in infant feeding practices between CMC (*n* = 47) and CMI (*n* = 275).

	**CMC**	**CMI**	***P***
	**(%)**	**(%)**	
The first liquid child was fed			<0.001
Breast milk	44.7	52.0	
Infant formula milk	23.4	45.5	
Plain water	17.0	2.2	
Sugar water	12.8	0.4	
Herbal preparation	2.1	0.0	
When was breast milk first introduced?			0.002
≤1 h after childbirth	17.0	35.4	
2–24 h after childbirth	48.9	25.0	
≥2 days after childbirth	21.3	16.0	
Never	12.8	23.5	
When was water first introduced?			
<1 week	84.8	59.3	0.003
1 week – 3 months	8.7	30.8	
≥4 months	6.5	9.9	
When were other non-milk liquids (juice, tea, etc.) first introduced?			
<4 months	62.2	19.8	<0.001
4–6 months	20.0	43.7	
>6 months	17.8	36.5	
When was infant formula first introduced?			
<4 months	46.8	83.5	<0.001
≥4 months	27.7	12.5	
Never	25.5	4.0	
When was cow's milk first introduced?			
<12 months	31.8	8.5	<0.001
≥12 months	68.2	91.5	
During the milk-fed period, child was fed			ns
On demand	36.2	39.1	
According to a scheduled routine	63.8	60.9	
First time of solid foods introduction			0.003
<4 months	41.3	19.3	
4–6 months	58.7	78.1	
>6 months	0.0	2.6	
What type of solid food was first introduced?			0.009
Egg yolk	34.8	19.2	
Fruit	21.7	10.0	
Vegetables	2.2	2.2	
Biscuit	0.0	0.4	
Commercial baby food	17.4	45.8	
Traditional Chinese infant staple (e.g., rice porridge/soft rice)	13.0	11.1	
Infant cereal	10.9	11.4	
In which month after birth was the following food first introduced?	(Mean ± S.D.)	(Mean ± S.D.)	
Rice porridge	5.76 ± 2.19	6.26 ± 2.22	ns
Vegetable	5.75 ± 2.45	6.16 ± 2.24	ns
Fruit	4.63 ± 1.92	5.25 ± 1.92	ns
Egg yolk	4.90 ± 2.69	5.78 ± 2.45	0.030
Meat/fish	7.47 ± 6.73	7.34 ± 2.72	ns
Normal family meal	12.39 ± 6.37	12.60 ± 5.14	ns

*CMC, Chinese mother gave birth in China; CMI, Chinese mother gave birth in Ireland*.

*Significance of relationships was calculated by Pearson Chi-squared tests for categorical variables and independent sample t-tests for continuous variables (average timings of solid introduction). Relationships were deemed to be statistically significant when P < 0.05. ns: no significant difference*.

Over two-fifths of CMC gave their babies complementary foods before 4 months, compared with less than one-fifth of CMI. For the first solid foods introduced, egg yolk (34.8%) and commercial baby foods (45.8%) were commonly used by CMC and CMI, respectively. The proportions of mothers using traditional Chinese infant staples (e.g., rice porridge/soft rice) were similar between CMC and CMI. For the timing of solid introduction, fruit and egg yolk were introduced earlier (4–5 months), followed by rice porridge and vegetable (5–6 months), and meat/fish (7 months). A normal family meal was generally introduced after 1 years of age. No significant differences in the time of first introduction between CMC and CMI were found among most of the foods, except that egg yolk was found to be introduced earlier among CMC (4.90 ± 2.69, months) than CMI (5.78 ± 2.45, months) (*P* = 0.030) (Table 2).

Multivariate analyses revealed that CMI was less likely breastfeed for 4 months and above (*OR* = 0.181, 95% CI: 0.055–0.595, *P* = 0.005), introduced water to their child within 1 week after childbirth (*OR* = 0.267, 95% CI: 0.073–0.979, *P* = 0.046), but more likely to introduce infant formula within 4 months after childbirth (*OR* = 7.489, 95% CI: 2.242–25.019, *P* = 0.001), in comparison to CMC, after controlling for potential confounders (Table 3).

**Table 3:** Multivariate analyses on infant feeding practices between CMC and CMI, after controlling for potential confounders.

	**CMC**	**CMI**	***P***
	**OR (95% CI)**	**OR (95% CI)**	
Breastfeeding duration			
>4 months vs. < 4 months	1	0.181 (0.055, 0.595)	0.005
The first liquid child was fed			
Breast milk vs. Others (Infant formula, Plain water, Sugar water, Herbal preparation)	1	1.959 (0.688, 5.582)	0.208
Infant formula milk vs. Others (Breast milk, Plain water, Sugar water, Herbal preparation)	1	2.247 (0.730, 6.918)	0.158
When was breast milk first introduced?			
<1 h vs. >1 h after childbirth	1	2.705 (0.787, 9.305)	0.114
When was water first introduced?			
<1 week vs. >1 week	1	0.267 (0.073, 0.979)	0.046
When were other non-milk liquids (juice, tea, etc.) first introduced?			
<4 months vs. >4 months	1	0.504 (0.158, 1.605)	0.246
When was infant formula first introduced?			
<4 months vs. >4 months	1	7.489 (2.242, 25.019)	0.001
When was cow's milk first introduced?			
<12 months vs. >12 months	1	0.974 (0.209, 4.541)	0.973
First time of solid foods introduction			
<4 months vs. >4 months	1	0.591 (0.158, 2.217)	0.436
What type of solid food was first introduced?			
Egg yolk vs. Other foods (fruits, vegetables, biscuit, commercial baby food, traditional Chinese infant staple, infant cereal)	1	0.958 (0.244, 3.770)	0.952
Commercial baby food vs. Other foods (egg yolk, fruits, vegetables, biscuit, traditional Chinese infant staple, infant cereal)	1	1.108 (0.903, 1.023)	0.859

*CMC, Chinese mother gave birth in China; CMI, Chinese mother gave birth in Ireland*.

*Maternal age at time of childbirth, mothers' duration in Ireland, child's order, child's age, maternal education level, marital status, and maternal birthplace were adjusted as in each regression model*.

### Factors influencing infant feeding practices of CMC and CMI

Over 83% of CMI delivered vaginally compared with only 68.1% of CMC. Most of the mothers roomed-in with their newborn during the first few days after childbirth, and CMC (44.7%) were more likely to bed-share with the newborn while CMI (70.4%) were more likely to sleep in a different bed. CMC and CMI reported similar sources of infant feeding information, including their own mother, doctors and other health professionals, friends, pamphlets or booklet, internet, etc. (data not shown), and a significantly higher proportion of CMC (48.9%) obtained the information from ante-natal/parent-craft classes, comparing with only 27.0% of CMI (*P* = 0.003) (Table 4). The husband was the most important source of family support in Ireland whereas the newborn's grandmothers played a more important role in China (*P* < 0.001). For breastfeeding mothers, CMC (47.5%) were more likely than CMI (22.1%) to receive breastfeeding support from their mother-in-law (*P* = 0.001). The majority of mothers went back to work or study before the first half year after childbirth but 41.4% of CMI stayed at home for at least 1 year (Table 4).

**Table 4:** Factors influencing infant feeding practices of CMC and CMI.

	**CMC**	**CMI**	***P*^†^**
	**(%)**	**(%)**	
Delivery mode^a^			0.025
Vaginal	68.1	83.2	
Caesarean	31.9	16.8	
During the first few days after birth, newborn and mother slept^a^			<0.001
In the same bed	44.7	24.8	
In the same room but in a separated bed	40.4	70.4	
In different rooms	14.9	4.7	
Obtained infant feeding information from ante-natal/parent-craft classes^a^	48.9	27.0	0.003
During the first month after birth, mother was looked after by^a^			
Nobody	0.0	12.7	0.004
Husband/partner	36.2	68.7	<0.001
Her mother	36.2	12.7	<0.001
Her mother-in-law	63.8	7.6	<0.001
How long after birth did mother go back to work/study?^a^			ns
≤6 months	57.4	41.8	
7–12 months	14.9	16.8	
>12 months, and never came back to work	27.7	41.4	
Reasons for choosing breastfeeding^b^			
Better for the baby	90.0	86.6	ns
More convenient and easier	55.0	30.2	0.003
Promote mother-infant bonding	72.5	51.5	0.015
Breastfed previous child/children	0.0	12.9	0.011
Planned breastfeeding duration^b^			0.010
<4 months	2.6	20.1	
4–6 months	28.2	37.0	
>6–12 months	59.0	37.0	
>12 months & the longer the better	10.3	5.8	
Mother-in-law supported breastfeeding^b^	47.5	22.1	0.001
Reasons for stopping breastfeeding^b^			
Formula feeding easier and more convenient	2.5	9.1	ns
Insufficient breast milk	32.5	53.4	0.024
Went back to work or study	35.0	27.9	ns
Mother thought it was the adequate time to stop	40.0	31.7	ns
Child was sent back China	0.0	1.9	ns
Methods used to increase breast milk supply^b^			
None	12.5	30.7	0.020
Traditional Chinese diet	87.5	68.3	0.013
Believed in the benefits of traditional Chinese diet^#^^a^	89.1	84.6	ns

*CMC, Chinese mother gave birth in China; CMI, Chinese mother gave birth in Ireland*.

† *Significance of relationships was calculated by Pearson Chi-squared tests. Relationships were deemed to be statistically significant when P < 0.05. ns: no significant difference*.

# Participants indicated “strongly agree/agree” (rather than “strongly disagree/disagree/don't know) with the statement: “Some traditional Chinese food can help to improve milk production.”

a *Question for the whole sample, including 47 CMC and 275 CMI*.

b *Question for mothers who breastfed their index child, including 41 CMC and 208 CMI*.

The benefits of breastfeeding to the babies were the main reasons given for breastfeeding, for both CMC and CMI. However, a significantly higher proportion of CMC breastfeeding mothers considered that breastfeeding promotes mother-infant bonding (72.5 vs. 51.5%) as well as being more convenient and easier (55 vs. 30.2%) than CMI breastfed mothers. About 13% of CMI breastfed because they had previous breastfeeding experience while none of the CMC indicated this reason (*P* = 0.011) (Table 4). “Formula feeding is more convenient” and “afraid baby being too attached” were indicated as the main reasons for mothers who had never breastfed (>25%, *P* > 0.05, data not shown). One-fifth of CMI planned to breastfeed for less than 4 months, compared with only 2.6% CMC. The majority of CMC planned to breastfeed for 6 to 12 months (59%) and another 10.3% planned to breastfeed for more than 1 year, while the corresponding figures for CMI were only 37.0 and 5.8%, respectively (*P* = 0.010). “Going back to work or study” as well as “it is the adequate time to stop” were the main reasons for breastfeeding discontinuation, among both CMC and CMI. About 2% of CMI breastfeeding mothers indicated sending the child back to China a few months after birth as one of the reasons for breastfeeding cessation. Over 53% of CMI breastfeeding mothers indicated insufficient breast milk as a reason, compared with 32.5% of CMC breastfeeding mothers (*p* = 0.024). To increase breast milk supply, 87.5% of CMC breastfed mothers consumed a special Chinese diet compared with 68.3% CMI (*P* = 0.013). A higher percentage of CMI did not use any methods to boost breast milk compared with CMC (*P* = 0.020) (Table 4).

## Discussion

A higher percentage of CMI than CMC were recruited and CMI were more likely to have been in Ireland for a longer period. This reflects the fact that Chinese women tended to have babies after settling down in Ireland. An overwhelmingly high proportion of CMC were first-time mothers (*n* = 45, 95.7%), while only 2 (4.3%) CMC were multiparous. In comparison, 37.8% of CMI (*n* = 104) were multiparous. Such differences between CMC and CMI might be contributed by a younger maternal age at time of childbirth of CMC compared with CMI. Moreover, some mothers who had given birth in China might give birth to another child (the index child in the study) in Ireland. In addition, the differences revealed might be explained by the “One child policy” in China between 1979 and 2015 (33). CMC were only allowed to have one child in China at the time when this study was conducted. Our sample profile reflects the actual profile of the Chinese immigration mothers. Similar profile has been revealed among Chinese mothers in Australia (19).

Migration did not appear to have an influence on breastfeeding initiation in this study population, as rates of breastfeeding initiation for both CMC and CMI were close to 85% (the Chinese national target rate in 2010) (15). The high initiation rate of CMI corroborates previous Chinese migration studies undertaken in Australia (5, 17, 19) and Canada (34) and previous observations that non-Irish nationals were more likely to breastfeed than Irish nationals (29, 35). However, our results contrasted with earlier studies in the UK (10) and the US (16) that reported low breastfeeding initiation rates among Chinese immigrants. Such differences may be due to migrant composition (dominated by Hong Kong mothers in the earlier studies) and the time frame of these surveys (15–20 years earlier).

Multivariate analyses indicated that breastfeeding duration of CMI was significantly shorter than that of CMC. The breastfeeding duration of CMI was shorter than the mean duration (7–9 months) reported in the majority of cities in China (15). The decline in breastfeeding duration among CMI concurs with a number of Chinese migration studies (17, 34, 36) and other Asian migration studies (7, 9, 37) in Western countries. The consistency in these findings suggests the important influence of migration on breastfeeding duration.

Comparison of factors influencing infant feeding practices between CMC and CMI provides possible explanations for the shorter duration. Firstly, as revealed in our multivariate analyses, a greater and earlier use of infant formula among CMI may contribute to the decline of breastfeeding duration. Secondly, cultural belief that the traditional post-partum diet is beneficial to breast milk production was prevalent in this study population. CMI were less likely to have consumed the traditional diet and more likely to have indicated “not enough breast milk” than CMC. It is possible that a lack of consumption of such a diet was linked closely to the perception of insufficient breast milk production, and in turn induced the reduction of breastfeeding duration. This issue was also reported by Rossiter (38) among Vietnamese immigrants in Australia. Thirdly, smaller numbers of CMI received support from their mothers and mothers-in-law, who in this culture are typically supportive of breastfeeding (12). Furthermore, owing to a lack of family support, some babies born in Ireland were sent back to China a few months after birth to be looked after by their grandparents. The separation of mother and child unavoidably contributed to shorter breastfeeding duration. Fourthly, few CMI obtained infant feeding information from ante-natal/parent-craft classes. A lack of appropriate infant feeding information was also reported by Goel et al. (10) who attributed this to Chinese mothers' language problems in the UK. In contrast, decline in breastfeeding duration was not found among Chinese mothers in Australia who reported receiving more breastfeeding support and assistance from health professionals than mothers in their home countries (19). This would therefore emphasize the importance of such support to increase duration rates. Fifthly, a common reason for CMI to breastfeed was they had breastfed before, however CMC breast-feeders mainly considered breastfeeding easy, convenient and promoting closeness to the baby. A higher percentage of CMI breastfeeding mothers considered formula feeding easier and more convenient. Such perceptual differences may lead to the difference in planned breastfeeding duration and in turn the actual duration (39). Finally, the planned duration of breastfeeding among CMI was significantly shorter than that of CMC, which may contribute to a shorter actual duration of CMI comparing to CMC. A number of studies have revealed that planned duration was the strongest predictor of actual breastfeeding duration (40–43). Such association could be explained by the Theory of Planned Behavior (TPB), which states that most actions of social relevance are under volitional control and that individual intention to perform an action is an immediate determinant of action (44).

The decline in breastfeeding duration among CMI did not appear to be associated with maternal employment, a negative factor reported consistently (1, 2). A high proportion of CMI stayed at home for at least 1 year post-partum or never worked anymore, which confirmed the low rate of participation of Chinese women in the labor market in Ireland (45). This finding was consistent with Vietnamese women having a baby in the US (37) but contrasts with Vietnamese women in Australia, who entered the workforce as soon as possible after confinement in an attempt to establish themselves and their families in the new country (46).

Differences in infant feeding patterns implicate environmental influence on infant feeding. Infant formula and commercial infant foods are popular in the Irish market. With the free provision of infant formula in the hospital, formula feeding has dominated in Ireland (47). Feeding the infants with water within 1 week after childbirth is prevalent among CMC compared with CMI. Such phenomenon could be explained by the Chinese traditional practices of early introduction to water. Moreover, our results indicate that complementary feeding with non-milk-liquids and solids before 4 months is widespread in China, because Chinese mothers are commonly concerned about the nutritional inadequacy of their breast milk to their infant (15). Rice porridge and other solid foods were introduced at a similar time between CMC and CMI, which suggested the retention of certain weaning practices among Chinese immigrants in Ireland. These findings concurred with earlier studies in Australia (19) and Canada (34) that infant practices among Chinese immigrants were subject to both Western and Eastern influences.

Prevalence of formula feeding among CMI and early introduction of water and complementary feeding among CMC suggests a need to promote exclusive breastfeeding for the first 6 months among the Chinese. Education to feed the newborns on demand (48) is needed since most CMC and CMI fed according to a scheduled routine.

The potential for recall bias should be acknowledged, especially in the case of older children (49). However, the data may be relatively accurate because 95.7% of CMC had only one child with an average age of 11.7 years, and studies have demonstrated that the duration of breastfeeding can be remembered accurately up to 10 years later (50). In addition, results of this study were specific to Chinese mothers in Ireland, limiting the study's generalizability to Asian mothers in other countries. Finally, owing to the reality discussed previously that CMC was dominated by first-time mothers, sample size and characteristics of CMC and CMI were unequal. To minimize bias, multivariate analyses have been performed to control for some potential confounders (e.g., child's order, child's age, maternal age at time of childbirth).

In conclusion, a sharp decline in breastfeeding duration was observed among CMI. The difference in infant feeding patterns between CMC and CMI suggests the importance of environmental influences on infant feeding practices. Breastfeeding support and education among Chinese in Ireland is needed, in particular to extend breastfeeding duration.

## Author contributions

QZ was responsible for the study design and development, data collection and analyses and drafting the manuscript. JK and KY were involved in study design and development, gave critical review and comments on this manuscript. All authors have read and approved the final version of the manuscript.

### Conflict of interest statement

The authors declare that the research was conducted in the absence of any commercial or financial relationships that could be construed as a potential conflict of interest.
